# Psoralen and Isopsoralen Ameliorate Sex Hormone Deficiency-Induced Osteoporosis in Female and Male Mice

**DOI:** 10.1155/2016/6869452

**Published:** 2016-04-30

**Authors:** Xiaomei Yuan, Yanan Bi, Zeman Yan, Weiling Pu, Yuhong Li, Kun Zhou

**Affiliations:** ^1^Institute of Traditional Chinese Medicine, Tianjin University of Traditional Chinese Medicine, Tianjin 300193, China; ^2^Tianjin State Key Laboratory of Modern Chinese Medicine, Tianjin 300193, China

## Abstract

Osteoporosis is a systemic skeletal disease, which is characterized by a systemic destruction of bone mass and microarchitecture. With life standard improved, the treatment of osteoporosis attracted more attention. The aim of this study is to verify the osteoprotective effect of psoralen and isopsoralen in females and males. Female and male mice were divided into 7 groups in this study: control group (sham-operation), model group (by ovariectomy or orchidectomy), positive control group (females given estradiol valerate; males given alendronate sodium), psoralen groups (10 mg/kg and 20 mg/kg), and isopsoralen groups (10 mg/kg and 20 mg/kg). After administration of psoralen and isopsoralen for 8 weeks, osteoporosis was ameliorated with increasing bone strength and improving trabecular bone microstructure as indicated by CT scan and pathology. Serum alkaline phosphatase (ALP), tartrate resistant acid phosphatase (TRACP), osteocalcin (OC), and C-terminal cross-linking telopeptides of type I collagen (CTX-1) were examined. Decreased TRACP and increased ALP/TRACP suggested restoring from bone destruction. These results suggest that psoralen and isopsoralen may be used as good natural compounds for the treatment of osteoporosis in males, as well as females.

## 1. Introduction

Osteoporosis is a common condition in the elderly, especially in postmenopausal women. It is characterized by a systemic destruction of bone mass and microarchitecture [1]. Bones provide structure, support, and protection to human body; they also contribute to movement. Osteoporosis leads to weakness and fragility of bone with risk of pathological fractures. What is more, increased mortality risk persisted for 5 years for all fractures and up to 10 years for hip fractures [2]. The medical and socioeconomic impact of osteoporosis increases further in the current aging population.

Osteoporosis can develop not only in women but also in men. In elderly women, bone mass losses and microarchitectural changes both are attributed to the occurrence of menopause. Although not experiencing menopause, men also present age-related acceleration in bone loss and microarchitecture deterioration [3]. The respective roles of estrogen receptor and androgen receptor are to be elucidated. Both estrogen receptor and androgen receptor are important in maintaining bone homeostasis [4]. One of the side effects of androgen deprivation therapy is osteoporosis, along with its high effectiveness in treating prostate cancer. And osteoporosis negatively affects the patient's quality of life [5]. It is estimated that one in five men suffer an event of osteoporotic fracture during their lifetime [6]. Most experimental studies observed the antiosteoporosis effect in ovariectomized animals, but as mentioned above, it is also important to observe their antiosteoporosis effects in males.

As an alternative treatment of osteoporosis and many other diseases, therapeutic effects of natural products derived from plants are research hot spots. Traditional Chinese Medicine Fructus Psoraleae is widely used in the treatment of osteoporosis. Psoralen and isopsoralen are the quality-control components of Fructus Psoraleae according to Chinese Pharmacopoeia. Recent studies have demonstrated psoralen, which is isolated from seeds of* Psoralea corylifolia *L., has antiosteoporosis effect in ovariectomy-induced osteoporotic rats via stimulating osteoblastic differentiation from bone mesenchymal stem cells [7]. Isopsoralen is the isomer of psoralen, whose antiosteoporosis effect is not clearly identified. Furthermore, it is not clear whether psoralen and isopsoralen have therapeutic effect on osteoporosis in male.

In this study, we demonstrated that psoralen and isopsoralen have significant osteoprotective effect in female and male mice with sex hormone deprivation. Bone strength increased and trabecular bone microstructure improved after administration of psoralen or isopsoralen for 8 weeks.

## 2. Materials and Methods

### 2.1. Drugs

Psoralen and isopsoralen, isolated from seeds of* Psoralea corylifolia* L., were purchased from Dalian Meilun Biotech Co., Ltd. (Dalian, China). Estradiol valerate tablet (Progynova) is product of Delpharm Lille S.A.S (France). Alendronate Sodium tablet is product of Merck Sharp & Dohme Italia PSA (Italy).

### 2.2. Animals and Treatment

ICR mice were purchased from Beijing HFK Bioscience Technology Co. Ltd. (Beijing, China). The mice were housed in the laboratory animal center of Tianjin University of Traditional Chinese Medicine at 18–22°C, humidity 55–65%. Mice eat standard diet and drink* ad libitum*. The animals experiments were approved by the Laboratory Animal Ethics Committee of Tianjin University of Traditional Chinese Medicine (permit number: TCM-LAEC 2015003).

The female mice were 8 weeks old in this experiment, and male mice were 10 weeks old. Female mice were ovariectomized and male mice were orchidectomized to establish experimental model of osteoporosis (a review about models of osteoporosis [8]). After 6 weeks, surviving mice were randomly divided into 6 groups: model group (females by ovariectomy or males by orchidectomy), positive drug group (females with estradiol valerate and males with alendronate sodium), psoralen groups (10 mg/kg and 20 mg/kg) and isopsoralen groups (10 mg/kg and 20 mg/kg), every group having 12 mice. On the other hand, control group includes 12 mice which underwent sham-operation. Medicines were administrated intragastrically once a day for 8 weeks. Mice in control group and model group mice were treated with water. Finally, the mice were anesthetized and sacrificed. Blood was collected to examine serum indicators, tibia was used to detect bone density and pathological analysis, and femur was used to test bone strength.

### 2.3. Measurement of Serum Indicators

According to manufacturer's protocol, serum alkaline phosphatase (ALP) and tartrate resistant acid phosphatase (TRACP) were measured using ALP and TRACP kits (Beyotime Institute of Biotechnology, Jiangsu, China). Serum osteocalcin (OC) and C-terminal cross-linking telopeptides of type I collagen (CTX-1) were measured using ELISA kits (Cloud-Clone Co., Ltd., Wuhan, China).

### 2.4. Bone Density Analysis by Micro-CT

Right tibia was preserved in 75% ethanol for 72 h, with ethanol being replaced once every 24 h. And then, tibia was preserved in anhydrous ethanol and bone structural indices and trabecular bone morphometry were performed using viva CT40 (SCANCO Medical AG, Zurich, Switzerland). Degree of anisotropy (DA) was the ratio of longest vector of mean intercept length (MIL) sensor over shortest vector of MIL sensor. Relative bone volume (BV/TV), trabecular number (Tb.N), trabecular thickness (Tb.Th), and trabecular separation (Tb.Sp) were calculated by plate model, and the scan region was below the epiphyseal growth plate of proximal tibia, extending about 1 mm towards the distal direction.

### 2.5. Histomorphological Analysis

Left tibia was fixed with 10% buffered formalin and decalcified with 8% formic acid and 8% oxalic acid and then embedded in paraffin to be sliced at 5 *μ*m. Sections were stained with hematoxylin and eosin (H&E). Histomorphological examination was performed by BX51 optical microscope (OLYPUS, Tokyo, Japan).

### 2.6. Bone Strength Analysis

After accomplishment of bone density analysis, right femur was used to test bone strength in mode 1 using YLS-16A small animal bone strength analyzer (Jinan Yiyan Technology Co. Ltd., Jinan, China), and the result is the femur maximum load capacity which indicates the maximum force (showed as gram) being applied to the femur until it was fractured.

### 2.7. Statistical Analysis

Data are expressed as mean ± SEM. Statistical analysis was performed according to one-way ANOVA analysis. The probability values of *p* < 0.05 were considered significant.

## 3. Results and Discussion

**Figure 1:**
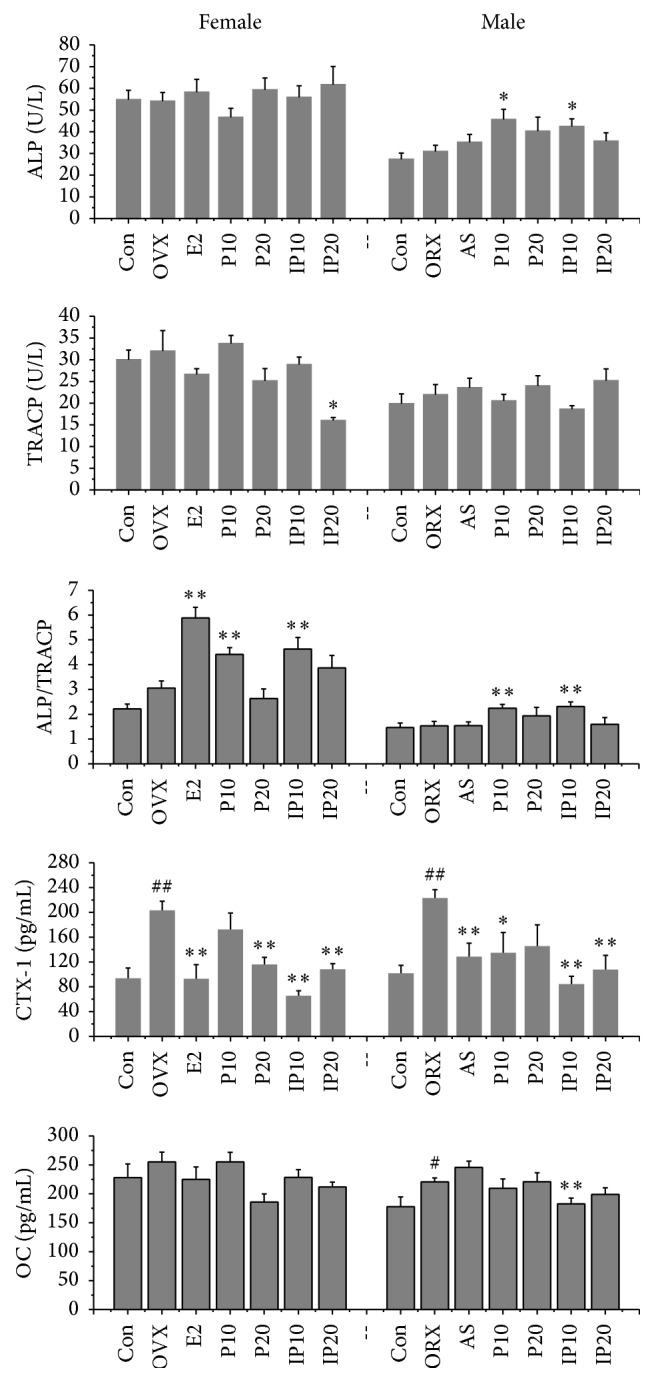
Effects of psoralen and isopsoralen on serum indicators. Con indicates control group; OVX and ORX both belong to model group; E2 indicates estradiol valerate group; AS stands for alendronate sodium group; P10 and P20 indicate psoralen 10 mg/kg and 20 mg/kg groups; IP10 and IP20 indicate isopsoralen 10 mg/kg and 20 mg/kg groups. ^#^ *p* < 0.05, ^##^ *p* < 0.01 compared with control group; ^*∗*^ *p* < 0.05, ^*∗∗*^ *p* < 0.01 compared with model group.

Bone is a dynamic organ that undergoes continuous remodeling while maintaining a balance between bone formation and resorption [9]. OC has routinely been used as a serum marker of osteoblastic bone formation and has been believed to play role in the bone matrix to regulate mineralization, but new genetic and pharmacologic evidence now points to a hormonal role for the protein [10]. The results showed that there were no significant differences in the increase of OC content in model groups or decrease in treatment groups. In addition, we employed serum ALP and TRACP as the markers of osteoblasts and osteoclasts activities. Although it was reported that psoralen could stimulate the osteoblastic differentiation from bone mesenchymal stem cells [7],* Psoralea corylifolia* was reported to be able to stimulate osteoblasts proliferation and differentiation in another study. This indicated that psoralen and isopsoralen present no significant effect on osteoblasts proliferation and ALP activity* in vitro* [11]. Serum ALP and TRACP had no significant difference between control group and model group. However, serum ALP increased in male mice treated with 10 mg/kg psoralen and isopsoralen while TRACP decreased in female mice treated with 20 mg/kg isopsoralen. Moreover, the values of ALP/TRACP significantly increased in female and male mice treated with 10 mg/kg psoralen and isopsoralen (Figure 1). These results suggested the high rate of bone formation compared to bone resorption in mice treated with psoralen and isopsoralen. CTX-1 is also a bone resorption marker like TRACP. The CTX-1 is small peptide fragments of degraded type I collagen that is secreted into the blood stream during bone resorption by osteoclasts because type I collagen accounts for more than 90% of the organic matrix of bone and is synthesized primarily in bone [12]. One study showed that a higher serum level of CTX-1 was associated with bone mineral density, and it is a simple approach to identify men with risk for osteoporosis [13]. Experimental results indicate that the serum CTX-1 of male and female model groups is two times higher than those of the control groups (*p* < 0.01), and CTX-1 decreased significantly after administration with psoralen or isopsoralen for 8 weeks.

**Figure 2:**
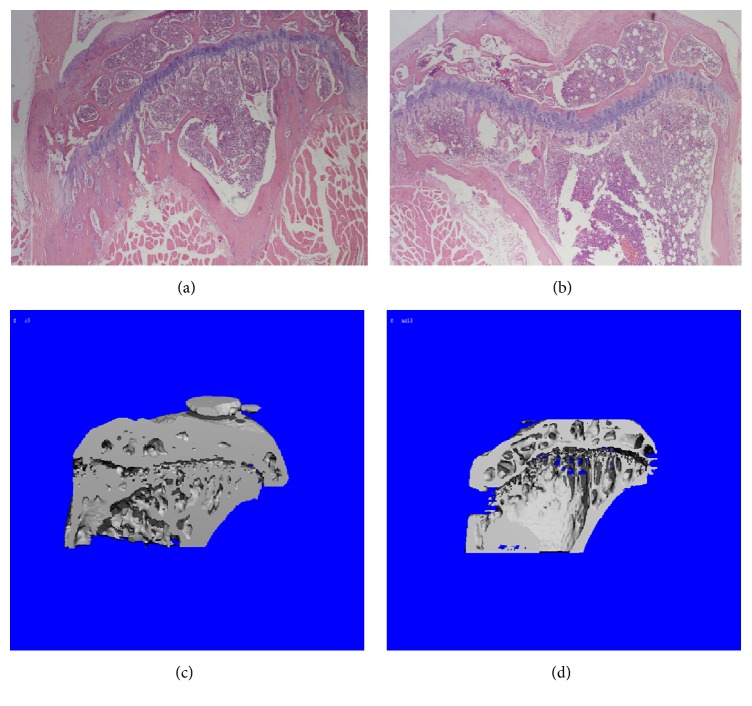
Bone microstructure changes of tibia after OVX or ORX. Histomorphological photomicrographs: (a) control mice, (b) model mice; The CT-scanning images: (c) control mice, (d) model mice.

Bone histomorphological photomicrographs and CT-scanning images show advantages in evaluating antiosteoporosis effect. As shown in Figure 2, there was significant difference between normal and abnormal groups. Trabecular bone loss can be observed in the mice of model group compared with control group, in either ovariectomized female mice or orchidectomized male mice. What is more, trabecular bone almost disappeared near the growth plate.

**Figure 3:**
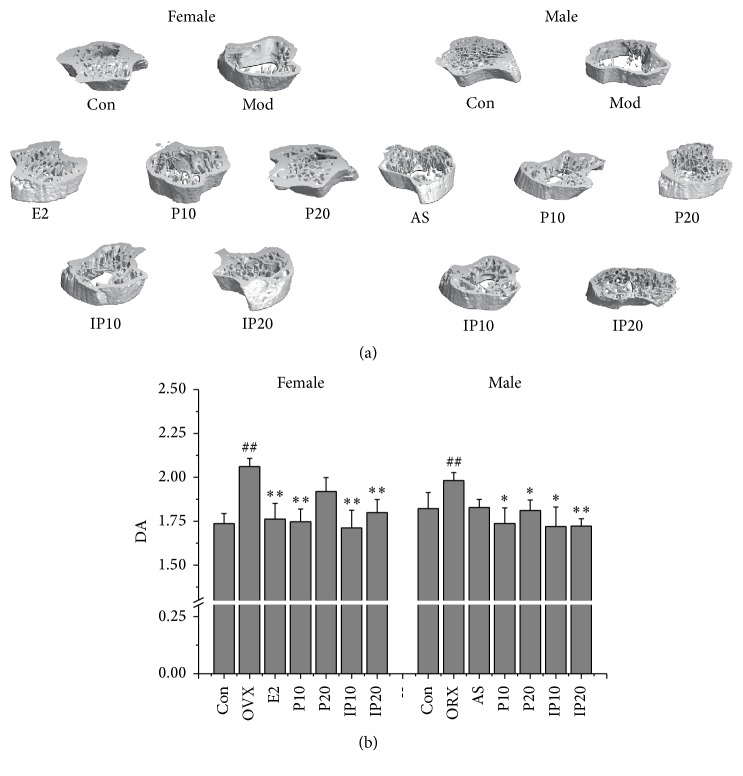
(a) The CT-scanning images. (b) Effects of psoralen and isopsoralen on DA. Con, control group; OVX and ORX both belong to model group; E2, estradiol valerate group; AS, alendronate sodium group; P10 and P20, psoralen 10 mg/kg and psoralen 20 mg/kg groups; IP10 and IP20, isopsoralen 10 mg/kg and isopsoralen 20 mg/kg groups. ^##^ *p* < 0.01 compared with control group; ^*∗*^ *p* < 0.05, ^*∗∗*^ *p* < 0.01 compared with model group.

Micro-CT can show high resolution in analyzing bone microstructure [14]. CT reconstruction was performed by means of a 3D filtered back-projection algorithm to retrieve the 3D bone structure image. Chappard et al. [15] demonstrated that juxtacortical trabeculae are less sensitive to aging and osteoporosis changes, and DA is a sensitive index to distinguish osteoporosis cases from control cases. In our study, trabecular bones of mice in model group were reduced (Figure 3(a)). DA was significantly greater than that of control group (Figure 3(b)). The DA of treatment groups, both in female and male mice, was reduced and close to normal.

**Figure 4:**
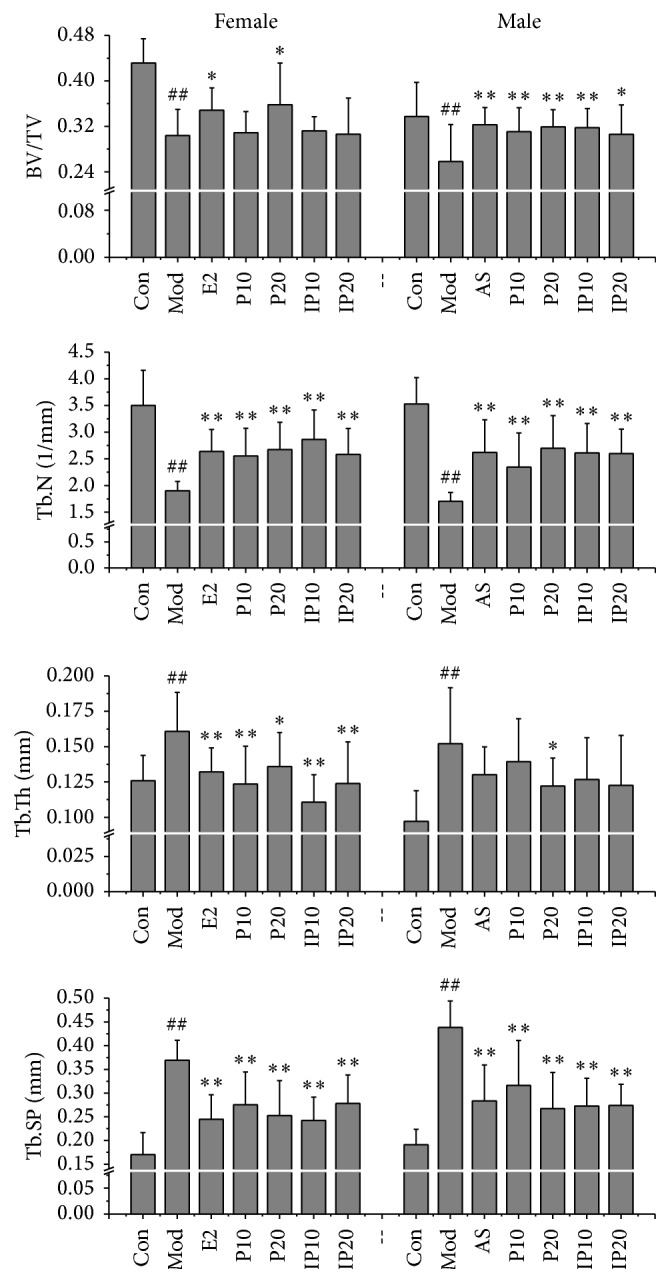
Effects of psoralen and isopsoralen on Conn.D, Tb.N, Tb.Th, and Tb.Sp (Con, control group; Mod, model group; E2, estradiol valerate group; AS, alendronate sodium group; P10 and P20, psoralen 10 mg/kg and psoralen 20 mg/kg groups; IP10 and IP20, isopsoralen 10 mg/kg and isopsoralen 20 mg/kg groups). ^##^ *p* < 0.01 compared with control group; ^*∗*^ *p* < 0.05, ^*∗∗*^ *p* < 0.01 compared with model group.

Micro-CT can not only provide three-dimensional microstructures of skeleton, but also show relative bone volume and bone trabecular thickness, and so forth. It is an accurate way to delineate the 3-dimensional architectural variables of trabecular bone and the physical properties of bone in a nondestructive way. 3D morphometric evaluation of trabecular region of interest (ROI) is superseded after setting up the scanning and analysis protocols to obtain the bone structural parameters results. Trabecular bone strength is relative to Tb.N, Tb.Th, and Tb.Sp. The severer the osteoporosis is, the greater the value of Tb.Sp is; then the distance between the trabecular bones will be farther. Tb.Sp in ovariectomized and orchidectomized mice increased twofold compared with those in normal mice (Figure 4). BV/TV is the percentage of trabecular bone area to reflect bone mass. This index is the most important objective one to evaluate the drug effects on bone mass. Antiosteoporosis drugs are generally able to increase the percentage of trabecular bone area. And lack of sex hormones can lead to the decrease in the number of trabecular bones, widened gap, and change of the rod of trabecular bone. In the current study, as show in Figure 4, we found that, compared with control groups, less bone volume fraction, less Tb.N, and more trabecular thickness were seen in model groups no matter male or female (*p* < 0.01), In male mice, the treatment of psoralen/isopsoralen increased BV/TV and Tb.N (*p* < 0.01). But in female mice, Tb.N and Tb.Th have improved, and BV/TV only treated with 20 mg/kg dose psoralen increased and the remaining three groups had no significant effect.

**Figure 5:**
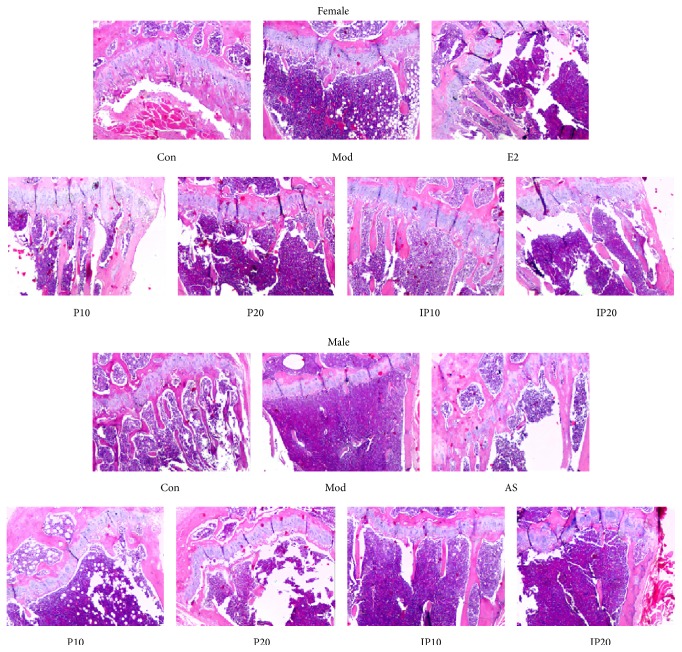
Bone histomorphological photomicrographs. Con, control group; Mod, model group; E2, estradiol valerate group; AS, alendronate sodium group; P10 and P20, psoralen 10 mg/kg and psoralen 20 mg/kg groups; IP10 and IP20, isopsoralen 10 mg/kg and isopsoralen 20 mg/kg groups.

Microstructure and function of bone tissue are closely related, and the changes in bone tissue microstructure have a significant impact on the biomechanics of bone. As seen in Figure 5, epiphyseal plate chondrocytes were columnar, arranged at the upper tibia, and with no cartilage cell necrosis area in each mouse of control group. Trabecular bone formation is more active, thick, and complete in control group. Trabecular bone formation was more active, thick, and complete, the morphological structure lined up tightly, the density and the area are normal, and the gap is smaller in control group. The bone trabecular number in model mice was significantly reduced, and trabecular bone gap increased. The number of transitional trabecular bones which were under the epiphyseal plate was reduced as well. Compared with model group, the trabecular numbers of psoralen/isopsoralen-treated mice were increased; intramembranous ossification and the trabecular bone area were also elevated. What is more, trabecular gap decreased, but still not as good as the control group.

**Figure 6:**
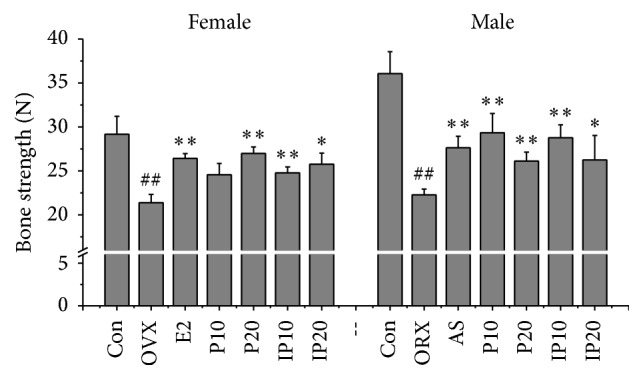
Effects of psoralen and isopsoralen on bone strength. Con, control group; OVX and ORX both belong to model group; E2, estradiol valerate group; AS, alendronate sodium group; P10 and P20, psoralen 10 mg/kg and psoralen 20 mg/kg groups; IP10 and IP20, isopsoralen 10 mg/kg and isopsoralen 20 mg/kg groups. ^##^ *p* < 0.01 compared with control group; ^*∗*^ *p* < 0.05, ^*∗∗*^ *p* < 0.01 compared with model group.

Bone strength is the skeleton to withstand the biggest load before fracture. It is affected by many factors, for instance, bone mass, bone structure and geometry, and internal bone quality. The purpose of the treatment of osteoporosis is to improve the bone strength, thus reducing the risk of fracture. The serious consequence of osteoporosis is fracture, and bone strength is the most important factor related to fracture risk. So the bone strength is important in evaluation of osteoprotective effect. Then, each animal's right femur was used to determine the bone strength as maximum antifracture capacity. Unsurprisingly, the results showed that bone strength of model group mice was significantly lower than that of mice in control group (*p* < 0.01), and bone strength increased after administration of psoralen and isopsoralen (*p* < 0.05) (Figure 6). This proved that psoralen and isopsoralen have the effect to protect osteoporotic fracture.

## 4. Conclusion

In summary, our findings showed that psoralen and isopsoralen both have the therapeutic effect in male mice, as well as in female mice. It may explain why Fructus Psoraleae can treat osteoporosis. However, these findings suggest that these two nature components may be used as good natural products candidate in treating osteoporosis in men.
